# Discovery and validation of an INflammatory PROtein-driven GAstric cancer Signature (INPROGAS) using antibody microarray-based oncoproteomics

**DOI:** 10.18632/oncotarget.1879

**Published:** 2014-03-31

**Authors:** Manuel Puig-Costa, Antonio Codina-Cazador, Elisabet Cortés-Pastoret, Cristina Oliveras-Ferraros, Sílvia Cufí, Sílvia Flaquer, Francesca Llopis-Puigmarti, Eulalia Pujol-Amado, Bruna Corominas-Faja, Elisabet Cuyàs, Rosa Ortiz, Eugeni Lopez-Bonet, Bernardo Queralt, Raquel Guardeño, Begoña Martin-Castillo, Josep Roig, Jorge Joven, Javier A. Menendez

**Affiliations:** ^1^ Department of General and Digestive Surgery, Dr. Josep Trueta University Hospital, Catalonia, Spain; ^2^ Girona Biomedical Research Institute (IDIBGI), Girona, Catalonia, Spain; ^3^ Metabolism & Cancer Group, Translational Research Laboratory, Catalan Institute of Oncology, Girona, Catalonia, Spain; ^4^ Unit of Clinical Research, Catalan Institute of Oncology, Girona, Catalonia, Spain; ^5^ Department of Anatomical Pathology, Dr. Josep Trueta University Hospital, Girona, Catalonia, Spain; ^6^ Medical Oncology, Catalan Institute of Oncology, Girona, Catalonia, Spain; ^7^ Unitat de Recerca Biomèdica (URB-CRB), Institut d'Investigació Sanitaria Pere i Virgili (IISPV), Universitat Rovira i Virgili; Reus, Catalonia, Spain

**Keywords:** Gastric cancer, inflammation, antibody arrays, cytokines, angiogenesis

## Abstract

This study aimed to improve gastric cancer (GC) diagnosis by identifying and validating an INflammatory PROtein-driven GAstric cancer Signature (hereafter INPROGAS) using low-cost affinity proteomics. The detection of 120 cytokines, 43 angiogenic factors, 41 growth factors, 40 inflammatory factors and 10 metalloproteinases was performed using commercially available human antibody microarray-based arrays. We identified 21 inflammation-related proteins (INPROGAS) with significant differences in expression between GC tissues and normal gastric mucosa in a discovery cohort of matched pairs (n=10) of tumor/normal gastric tissues. Ingenuity pathway analysis confirmed the “inflammatory response”, “cellular movement” and “immune cell trafficking” as the most overrepresented biofunctions within INPROGAS. Using an expanded independent validation cohort (n = 22), INPROGAS classified gastric samples as “GC” or “non-GC” with a sensitivity of 82% (95% CI 59-94) and a specificity of 73% (95% CI 49-89). The positive predictive value and negative predictive value in this validation cohort were 75% (95% CI 53-90) and 80% (95% CI 56-94), respectively. The positive predictive value and negative predictive value in this validation cohort were 75% (95% CI 53-90) and 80% (95% CI 56-94), respectively. Antibody microarray analyses of the GC-associated inflammatory proteome identified a 21-protein INPROGAS that accurately discriminated GC from noncancerous gastric mucosa.

## INTRODUCTION

The prognosis for most patients with gastric cancer (GC) is poor and has improved little over the past several decades [1]. Currently, GC prognosis is based on pathology (*i.e.*, histological type, invasion and metastasis), radiological imaging (for staging) and other clinical factors (age and comorbidity). However, these traditional clinicopathological factors have significant limitations, and major efforts are therefore being made to develop molecular signature-based methods to complement the traditional histopathological methods for diagnosis, classification and prognosis in GC [2-5]. Unfortunately, the current molecular biomarkers of clinical GC (*e.g.*, p27, cyclin E, E-cadherin, HER2, c-Myc and p53) lack the sensitivity and specificity required for screening an asymptomatic population for the purpose of early detection [6-8].

With the development of microarray technologies, it is not surprising that the majority of molecular signatures for GC have been derived from gene expression microarray studies [9-13]. Several studies have identified genetic markers or gene expression profiles capable of distinguishing normal from malignant gastric growth, and these markers can aid in the prognosis and estimation of survival rates for GC patients. However, these signatures often contain large numbers of genes, which reduces their application in daily clinical practice. Alternatively, other mainstream high-throughput proteomic profiling techniques include gel-based methods, gel-free mass spectrometry (MS)-based methods and surface-enhanced laser desorption/ionization (SELDI) time-of-flight (TOF) MS, and the application of these techniques in tissue-based clinical studies has the potential to provide efficient biomarkers for GC [14-20]. However, the requirement of sophisticated devices greatly limits their broad application in routine clinical practice. Moreover, technological limitations due to patient-to-patient variability and loss of signal from low-abundance proteins have negatively impacted the field of disease proteomics focused on separation techniques coupled with MS-based protein identification.

Routine proteomic testing in patients at risk for GC is not practical or realistic on a large scale. Therefore, other simple and inexpensive tests will be necessary for such purposes. The use of capture reagents such as antibodies in affinity proteomics has emerged as a new tool capable of gathering information on the global level in a high-throughput format using multiple versions of affinity reagents (*e.g.*, full-length antibodies, aptamers, affibody molecules, single-chain variable fragments of antibodies) and various capture formats (*e.g.*, planar arrays, beads, antibodies in the array format) [21,22]. Moreover, antibody microarray technology has rapidly evolved from proof-of-concept to state-of-the-art technology capable of targeting complex, nonfractionated protein samples. Therefore, antibody arrays represent a new paradigm for biomarker proteomics that has the potential to accelerate biomarker discovery and validation compared to traditional methods of proteomics [23-29]. Antibody microarray-based technology, which can simultaneously detect the expression levels of multiple proteins and can combine the advantages of the specificity of ELISA, sensitivity of enhanced-chemiluminescence (ECL) and high-throughput capacity of microspot, represents a promising tool for the field of oncoproteomics. One of the most important applications of such technology is the comparison of proteome expression signatures in cancerous *versus* normal samples. Indeed, an ever-growing number of publications have documented the suitability of sandwich-based antibody arrays; these arrays are the most common of the antibody arrays used for protein detection and can characterize differential protein expression patterns using various sample types including serum, plasma, cell conditioned media, cell and tissue lysates, cerebrospinal fluid, urine, abscess fluid, sputum, breath condensates, saliva, tears, prostatic fluids, milk, colostrum, *etc*.

We hypothesized that antibody microarray analyses using whole-tumor samples as a starting material for protein profiling might provide a functional perspective to the view that GC emerges from active inflammatory cross-talk between tumor cells and the surrounding stroma. Thus, the interactions in the GC microenvironment should produce inflammation-associated proteomic profiles able to specifically identify the absence/presence of tissue malignancy [30-35]. To test the hypothesis that pathological processes causally linking inflammation with GC would produce disease-specific molecular changes during cancer development in the gastric mucosa, we herein applied the innovative, simple, flexible and cost-effective antibody-based protein array system. The aim of this study was to identify a unique “oncoproteomic signature” in a pilot study for biomarker discovery in patients with GC. We focused specifically on secreted signaling proteins including cytokines, angiogenic factors, growth factors, inflammatory factors and metalloproteinases, as these factors constitute the primary means of communication between cells in our body. We examined 120 cytokines, 43 angiogenic factors, 41 growth factors, 40 inflammatory factors and 10 metalloproteinases simultaneously in matched pairs of tumor/normal gastric tissues using commercially available Human Antibody Arrays (RayBiotech, Inc.). This system enabled the robust and accurate identification of more than 250 proteins in an inexpensive fashion. Importantly, the experiments were performed in a general laboratory setting without any specialized equipment or special training. Using this approach, we successfully identified a signature of 21 proteins for discriminating GC from noncancerous gastric mucosa with high sensitivity and specificity; we termed this signature INPROGAS (Inflammatory PROtein-driven GAstric cancer Signature).

## RESULTS

### Supervised proteomic analysis identified differentially expressed proteins in GC

**Figure 1:**
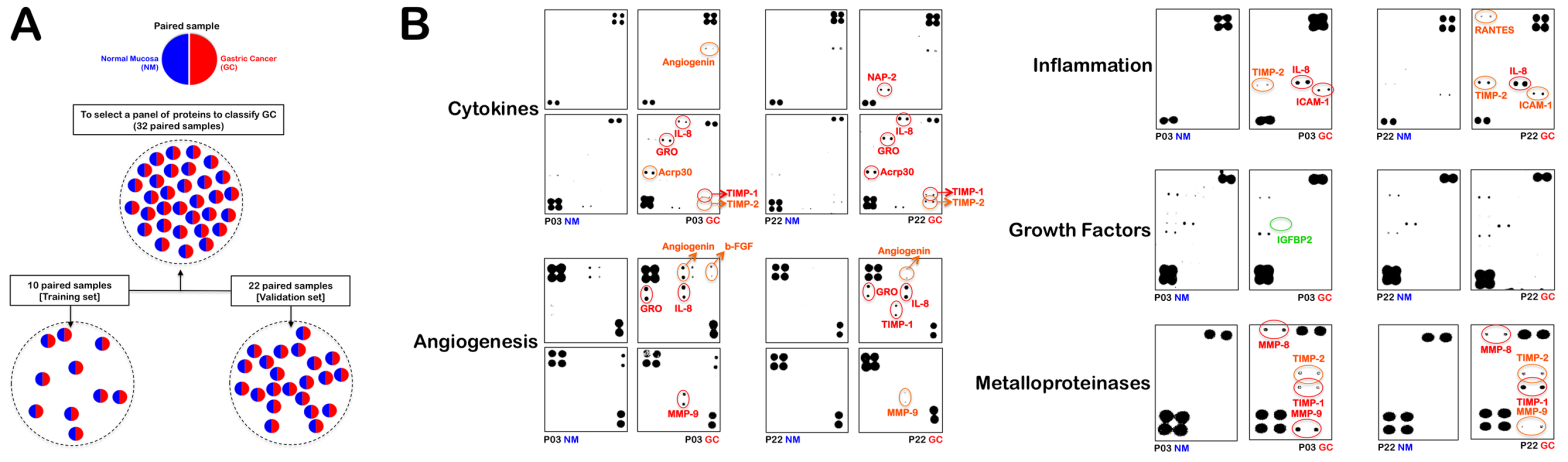
INPROGAS: Study outline and representative antibody-based array chips A. Informed consent was obtained from all human subjects according to the ethics committee guidelines at the Hospital Dr. Josep Trueta, Girona (Spain). A total of 32 paired GC/non-GC samples were separated into a training set (n=10) and a validation set (n=22), as indicated. B. This figure shows antibody-based array chips encompassing 120 cytokines, 43 angiogenic factors, 41 growth factors, 40 inflammatory factors and 10 metalloproteinases in duplicates probed with whole lysates from paired GC and non-GC mucosae in patients #3 and #22 (NM: Normal mucosa; GC: Gastric carcinoma). The membranes were treated with antibody cocktails, developed by an ECL kit and exposed to an X-ray film as described in the “Materials and Methods”. The intensity of each signal was evaluated photometrically using integrator software and normalized to the background noise in each spot relative to the negative controls. The spot intensities of each protein in replicates were then merged and expressed as a mean value relative to the average signals of the positive controls (membrane-bound biotin-conjugated antibodies) on the array chip analyzed for each experimental (GC) and control (NM) paired group.

**Figure 2:**
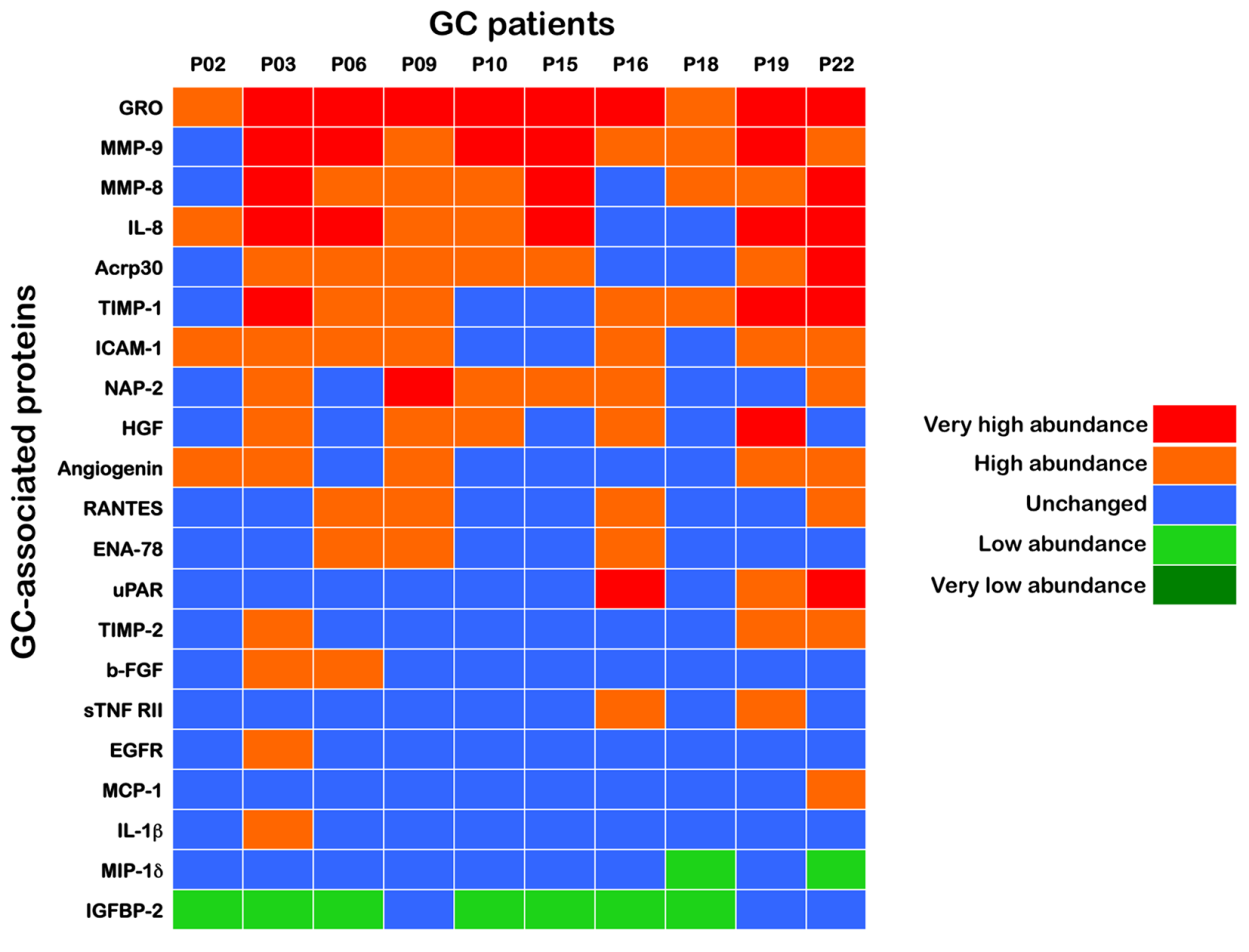
INPROGAS: A 21-protein signature that discriminates GC from noncancerous gastric mucosa Upon calculation of fold-changes for expression in GC relative to the matched non-GC sample, all proteins that were significantly expressed in a given GC tissue were arbitrarily placed into several “expression groups” based on their intensities relative to the paired non-GC tissue. We analyzed normalized array measurements in the training set to discover differences in protein abundance between samples of GC and those of non-GC to generate a signature of the inflammatory proteome (INPROGAS). Patient data were arranged in columns, and the proteins are listed in rows. Red shades, very high abundance (10-fold and over); orange shades, high abundance (3-fold to < 10-fold); blue shades, no-change (< ±3-fold); light green, low abundance (-3-fold to < -10-fold); dark green, very low abundance (-10-fold and under).

A supervised approach has the advantage of identifying proteins whose expression levels best correlate with clinical data. Therefore, we initially grouped a training set of paired samples (n=10) consisting of GC tissues and their corresponding adjacent, non-GC sections (Fig. 1A). To ensure the identification of a robust set of proteins differentially expressed in GC tissues, we used an analytic workflow with two stringent criteria. First, to minimize the individual differences among the patients' samples, equal amounts of protein from each sample, either the GC or the adjacent tissue, were applied to several microarrays of antibodies strictly in parallel. Second, following processing with antibody-based microarrays of the pairs of samples according to their known “GC” and “non-GC” phenotypic status, the ratio of the GC sample/non-GC sample cell lysates for each of the > 250 antibodies on the arrays was calculated to determine a unique value for the specific protein. The inflammatory proteome was then screened for proteins that were significantly altered from the non-GC spot intensities in the paired GC sample (either upregulated or downregulated; Fig. 1B) from each patient. All of the proteins that were significantly expressed in a given GC tissue were arbitrarily placed into the following groups based on their intensities relative to the paired non-GC tissue: very high abundance (10-fold and over), high abundance (3-fold to < 10-fold), no-change (< ±3-fold), low abundance (-3-fold to < -10-fold) and very low abundance (-10-fold and under). With this 2-step strategy, we identified 21 differentially expressed proteins in at least one of the paired samples in the training set, including 19 upregulated (GRO, MMP-9, IL-8, MMP-8, TIMP-1, Acrp30, ICAM-1, NAP-2, Angiogenin, HGF, b-FGF, RANTES, ENA-78, uPAR, sTNF RII, TIMP-2, EGFR, MCP-1 and IL-1β) and 2 downregulated (MIP-1δ and IGFBP-2) proteins in GC tissues. These identifications were grouped in the heat map shown in Fig. 2.

### INPROGAS: An INflammatory PROtein-driven GAstric cancer Signature

**Figure 3:**
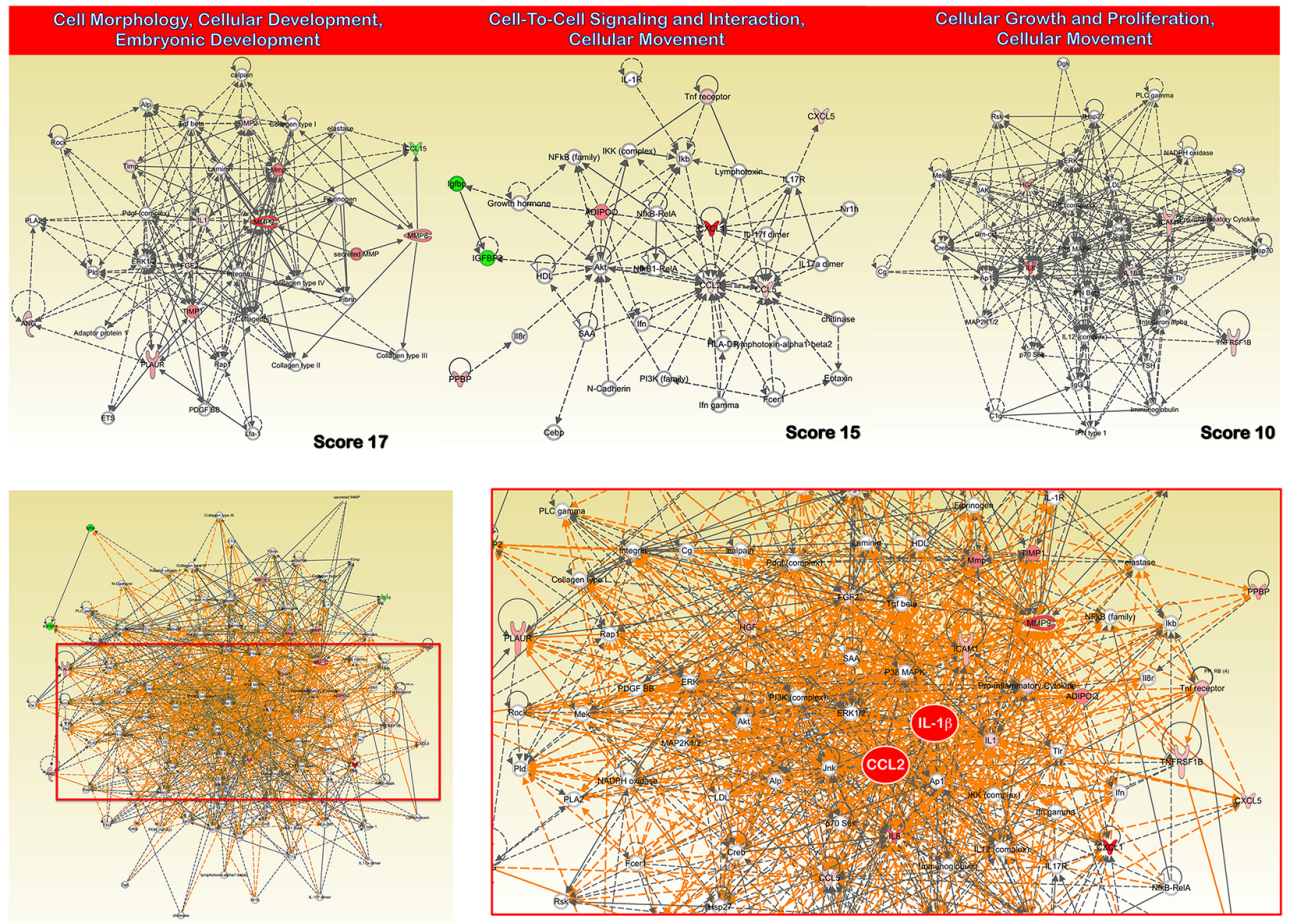
INPROGAS: a functional analysis Network analysis of differentially expressed proteins included in INPROGAS. A dataset containing the differentially expressed biomarkers in GC tissues (called the *focus molecules*, n=21) was overlaid onto a global molecular network developed from information contained in the IPA Knowledge Base. Networks of these focus molecules were then algorithmically generated based on their connectivity. *Top.* The figure shows the networks with the 3 highest IPA scores (a composite measure indicating the statistical significance of the interconnection between the molecules depicted in the network). The focus molecules are colored according to the gene expression (fold change) value; red gene symbols indicate upregulation, and green gene symbols indicate downregulation. The nodes are displayed using various shapes that represent the functional class of the gene product. Edges with dashed lines indicate indirect interactions, while continuous lines represent direct interactions. *Bottom*. Merged network combining major signaling networks depicted in top panels associated with the proteins included in INPROGAS.

**Figure 4:**
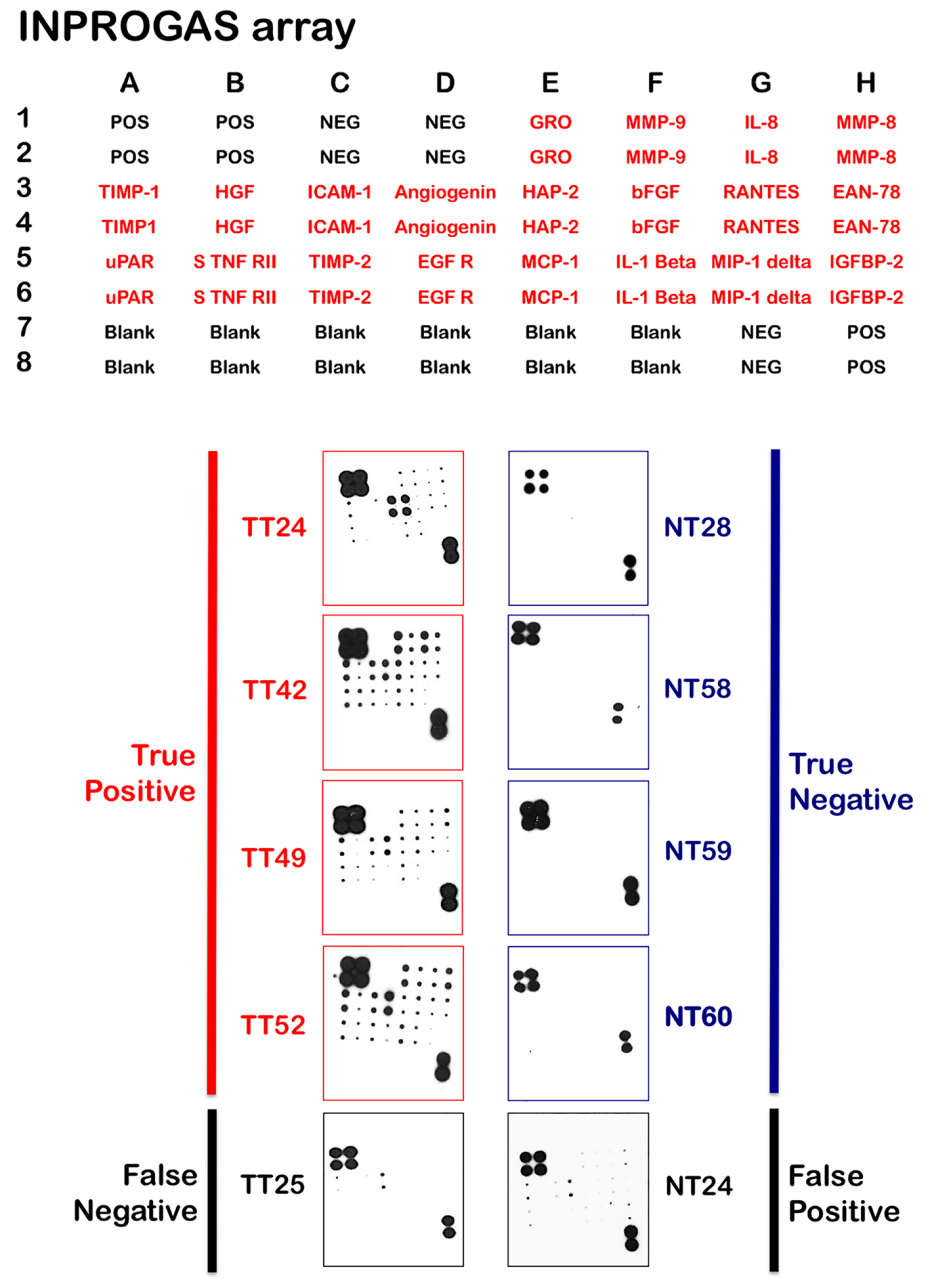
Classification and prediction of GC diagnosis using INPROGAS A. This figure shows antibody-based array INPROGAS chips for assessing the performance of the in the classification of unknown samples. B. The INPROGAS predictors identified in the training set were used for GC and non-GC class prediction in a blinded test set including 22 paired samples. This figure shows representative antibody-based array chips encompassing the 20 INPROGAS predictors (Acrp30 was excluded from the INPROGAS chip due to technical issues) in duplicates probed with whole lysates from non-tumor tissue (NT) and tumoral tissue (TT). The figure shows several representative images of tissues catalogued as true positive, true negative, false negative, and false positive.

When the 21 proteins with highly significant differences in expression between GC and non-GC tissues were assigned into categories based on Gene Ontology (GO) Consortium-defined *molecular function*, more than one-third (36%) of the proteins demonstrated cytokine/chemokine activity. When the proteins were classified based on GO Consortium-defined *biological process*, more than one-third (35%) of the proteins were involved in inflammatory/immune and chemotactic responses. Next, to reveal the key signaling pathways or networks related to the set of biomarkers identified in the training set, we imported the list of these 21 proteins into the IPA software (Fig. 3). According to the IPA knowledge base, 3 major signaling networks comprised of 35 nodes each were associated with this set of proteins (Fig. 3, *top panels*). Network 1 (cell morphology, cellular development, embryonic development) included 8 out of the 21 differentially expressed proteins (P-score=17) and mainly involved MMP- or tissue inhibitor of metalloproteinase (TIMP)-associated signaling. Network 2 (cell-to-cell signaling and interaction, cellular movement) included 7 out of the 21 differentially expressed proteins (P-score=15) and mainly involved MCP-1 (CCL2)- and CCL5-associated signaling. Network 3 (cellular growth and proliferation, cellular movement) included 5 out of the 21 differentially expressed proteins (P-score=10) and mainly involved interleukin (IL)-8- and IL-1-associated signaling. The pro-inflammatory action of the chemokines MCP-1 and IL-1 was central in a merged network combining the top 3 signaling networks with the highest IPA scores (Fig. 3, *bottom panels*). When we overlaid “diseases and disorders”, “molecular and cellular functions” and “physiological system” onto the merged core networks in IPA, 19 proteins were associated with the “inflammatory response” (*p* value = 6.98E-21), 20 proteins were associated with “cellular movement” (*p* value = 1.77E-23), and 18 proteins were associated with “immune cell trafficking” (*p* value = 1.77E-23), respectively. We termed this signature of 21 predictors that could discriminate GC from noncancerous gastric mucosa “INPROGAS” (INflammatory PROtein-driven GAstric cancer Signature; Fig. 4, *top panel*)

### Performance of INPROGAS in the classification of unknown gastric samples

Unlike gene expression profiles, in which the numbers of features vastly outnumber the size of the sample cohort, the limited number of features included on a protein array notably reduces the risk of over fitting when correlating expression with diagnosis. Nevertheless, we sought to validate the performance of INPROGAS in an independent sample cohort and performed GC profiling on a new set of 22 pairs of samples. None of these samples were included in the training set. Thus, to assess the predictive performance of INPROGAS in the classification of unknown samples, we performed a prediction for “GC” or “non-GC” phenotype (a so-called “2-class” prediction) in a blinded set containing paired samples of GC and normal mucosae obtained from 22 patients. For each sample, INPROGAS was performed, and samples were classified into “GC” or “non-GC” groups according to the discovery of positive biomarkers (positivity for at least 80% of the INPROGAS biomarkers was considered a “GC” identification) (Fig. 4, *bottom panel*). Using this expanded independent validation cohort, INPROGAS classified gastric samples as “GC” or as “non-GC” with a sensitivity of 82% (95% CI 59-94) and a specificity of 73% (95% CI 49-89) (Table 2). Moreover, INPROGAS demonstrated a high positive predictive value (75%; 95% CI 53-90) and negative predictive value (80%; 95% CI 56-94) in the validation cohort.

## DISCUSSION

Rudolph Virchow first suggested a causal link between inflammation and cancer in 1863 when he demonstrated the presence of leucocytes in neoplastic tissue. Although Virchow's original hypothesis was repeatedly revisited and there were ample data corroborating inflammation-mediated oncogenesis, inflammation was not recognized among the 6 *bona fide* hallmarks of cancer in a seminal contribution by Hanahan and Weinberg more than a decade ago [36]. Interestingly, recent years have seen a renaissance of the inflammation-cancer connection, leading to a generally accepted paradigm for inflammation in tumorigenesis, *i.e.*, an inflammatory microenvironment is an essential component of all tumors, including GC [37-44]. We now acknowledge that aberrant proliferation is insufficient to cause cancer, which additionally requires a cancer-promoting microenvironment rich in factors that support cellular survival, growth and angiogenesis. In fact, many cancer-related cytokines, chemokines, metalloproteinases, growth factors and angiogenic factors are produced not only by the tumor cells themselves but also by activated stroma and immune cells associated with tumors. All of these factors that accumulate *in situ* during chronic inflammation not only exert profound effects on (transformed) epithelial, endothelial and mesenchymal cells but also recruit immune cells. These findings highlight the close parallels between tumor initiation and wound inflammation (*i.e.*, “cancers as wounds that do not heal”).

The inflammatory mediators produced by immunocompetent cells and cancer cells can directly stimulate carcinogenesis (*e.g.*, *via* the induction of genetic instability in the epithelium of the human stomach), cancer proliferation, angiogenesis, cell adhesion, migration and invasion. In addition, the tumor microenvironment contributes to the systemic anti-inflammatory state associated with cancer (*i.e.*, inflammatory cytokines and chemokines leaking into the systemic circulation are likely to desensitize circulating leukocytes), actively promotes the tumor cells' ability to subvert the host's anti-tumorigenic innate and adaptive immune responses and indirectly leads to cancer progression [37-44]. Increasing evidence supports the hypothesis that the seventh hallmark of cancer, *i.e.*, cancer-related inflammation, is a key component of GC formation and progression. Our study hypothesized that processes such as aberrant cell growth, cell invasion, alterations in immune system function and the inflammatory response actively generate an altered or unbalanced stoichiometry of numerous molecules (*i.e.*, growth factors, pro-inflammatory molecules, cytokines, metalloproteinases and angiogenic/lymph angiogenic factors) in the tumor microenvironment compared to the “normal” microenvironment. We further speculated that these qualitative and/or quantitative changes could be characterized in the form of proteomic profiles that could differentiate GC from non-GC in the human stomach mucosae. The description of proteomic profiles specifically representative of the molecular interactions that occur in the GC tumor microenvironment may significantly increase the specificity and sensitivity provided by existing GC diagnostic markers, especially during early disease stages. Our current study confirmed that antibody-based microarray analyses of complex proteomes in gastric tissue-based studies are useful tools to define GC disease-associated protein signatures. Moreover, our proteomic profiling using whole-tumor samples supported the view that GC emerges from active cross-talk between tumor cells and the surrounding stroma, as many of the differentially regulated proteins were pro-inflammatory chemokines and MMPs.

A large number of the GC samples included in the training set revealed a role for the chemokine GRO/CXCL1, which plays a key role in inflammation, immunity, angiogenesis and cell movement [32, 45, 46]. In addition, MMP-9 and MMP-8, which act on pro-inflammatory cytokines, chemokines and other proteins to regulate varied aspects of inflammation and immunity [47, 49], showed a high prevalence among GC samples. Very high incidence rates were also observed for the pro-inflammatory factor IL-8, which has an established role in the chronic inflammation that underpins the development of a number of human cancers [50-52]. Moreover, TIMP-1, which has recently emerged as an important multifunctional protein capable of regulating inflammation, also reached a high prevalence among GC samples [53-55]. Paradoxically, Acrp30/adiponectin, an adipokine regulating glucose and lipid metabolism with well-known anti-inflammatory properties [56, 57], was found to be upregulated in a significant number of GC samples. Adiponectin has been shown to have antiproliferative effects on GC, and adiponectin expression is inversely correlated with clinical staging of the disease [58, 59]. Nevertheless, it should be noted that due to problems with combinatorial optimization during the design and production of the INPROGAS signature by RayBio Inc., the Acrp30/adiponectin marker was not included in custom microarrays. Therefore, the actual impact of Acrp30/adiponectin on GC diagnosis was not assessed in the current study. Similarly, one of the 21 predictors included in the INPROGAS signature was hepatocyte growth factor (HGF). HGF is a potent angiogenic factor that stimulates growth and motility of endothelial cells and has potent anti-inflammatory effects in multiple animal models of disease in various organs; in particular, HGF functions by suppressing NF-κB and downstream endothelial inflammation [60-62]. However, whether adiponectin and/or HGF act to protect the organism from systemic inflammation as part of the paradoxical local inflammation and systemic anti-inflammation during the development of GC remains to be answered in forthcoming studies. A high incidence rate was also found for intracellular adhesion molecule-1 (ICAM-1), a key contributor to vascular inflammation, as ICAM-1 ligation produces pro-inflammatory effects such as inflammatory leukocyte recruitment [63, 64]. In addition, angiogenin, a heparin-binding 14-kDa plasma protein that has been demonstrated to stimulate angiogenesis and is induced by pro-inflammatory cytokines to mediate local inflammation [65-67], was found to be significantly upregulated in GC samples. High incidence rates were also observed for the CXC chemokine *neutrophil-activating peptide-2* (NAP-2), a chemoattractant that is rapidly generated within the vasculature early during inflammation and potently induces effector functions in neutrophils, such as chemotaxis and degranulation [68, 69]. The platelet-derived pro-inflammatory chemokines RANTES and ENA-78 were found to be significantly co-upregulated among GC samples, and significant incidence rates were also detected for the urokinase-type plasminogen activator receptor (uPAR). uPAR expression is elevated during inflammation and tissue remodeling and in many poor-prognosis human cancers [70-72]. The anti-angiogenesis and anti-inflammatory factor TIMP-2 was also significantly upregulated in GC samples [73].

In contrast, other biomarkers included in the INPROGAS signature displayed lower incidence rates, including the cytokine inhibitor soluble tumor necrosis factor receptor II (sTNFRII) and the epidermal growth factor receptor (EGFR). EGFR is a signaling hub for an increasing list of growth factors, cytokines and inflammatory mediators that connects the inflammatory reaction to tumor development [74, 75]. Basic fibroblast growth factor (bFGF) is known to potentiate leukocyte recruitment to sites of inflammation by enhancing endothelial adhesion molecule expression [76]. The pro-inflammatory cytokine IL-1β is a key component of the multiprotein inflammasome complexes [77, 78]. Monocyte chemoattractant protein (MCP)-1 is a key chemokine of the C-C type that recruits circulating monocytes to sites of inflammation [79, 80], and MIP-1δ (CCL15) is a member of the macrophage inflammatory protein (MIP) family of CC-type chemokines that are mainly produced by leukocytes after exposure to inflammatory cytokines [81]. MIP-1δ play a major role in the recruitment of immune cells to sites of injury or infection and was found to be downregulated in 20% of the training set samples. Of note, in 70% of the training set samples, the binding protein for insulin-like growth factor (IGFBP)-2 was underexpressed. IGFBP-2 is crucial for modulating the levels of the IGF-1R ligand IGF-2, and loss of this regulatory protein leads to an increased availability of IGF-2 and thus constitutive activation of IGF-1R [82, 83]. The IGF-1R signaling pathway is involved in the carcinogenesis of GC through inhibiting cell apoptosis [84]. In the complex inflammatory scenario captured by the INPROGAS signature, it is reasonable to suggest that the local inflammatory network in GC is determined according to the expressed inflammation-related proteins, inflammatory receptor expression patterns and relative concentrations of pro- and anti-inflammatory biomarkers. Thus, the net inflammatory environment likely fluctuates during various stages of GC development.

Early diagnosis is likely to improve the outcome and prognosis of most solid tumors, including biologically aggressive, chemotherapy-refractory GC. Moreover, there is an urgent need to develop new approaches to detect and measure biomarkers in tissues and/or the blood because these markers could lead not only to early detection of GC but also to improved targeted treatments for GC patients. In this regard, because the discovery and validation of GC-associated proteomic profiles could transcend the problems of tumor heterogeneity and population dynamics, there is an urgent need to develop and validate new, simple, flexible, effective and highly sensitive proteomic analysis techniques. New techniques will also allow for the simultaneous analysis of several biomarkers in a single assay in a low-cost format for proper carcinoma diagnosis and/or staging during routine hospital practice. Here, we demonstrated that low-cost proteomic analysis using antibody-based protein microarrays represents a useful new tool for the routine early detection, diagnosis and perhaps therapeutic intervention of GC in hospitalized patients. To elucidate the core molecular networks underlying INPROGAS, the 21 biomarkers were analyzed using the Ingenuity Knowledge Base, which consists of expert-curated molecular interactions. By merging the three INPROGAS-associated core networks, we identified MCP-1 and IL-1β as hub proteins related to the “inflammatory response”, “cellular movement” and “immune cell trafficking” biofunctions. The IPA Knowledge Database further suggested that the involvement of these signaling networks could be essential for cancer development in the human stomach. Because perturbations of these hub proteins have functional effects on GC, small molecule inhibitors of nodes in INPROGAS-related networks may lead to dynamic changes in protein expression. This possibility could open new avenues for the manipulation of cytokine expression and function in cancer immunotherapy for GC.

In summary, we measured the relative abundance of more than 200 signaling proteins in an initial set of samples from 10 matched pairs of tumor/normal gastric tissues and found significantly different expression patterns of 21 proteins, which we have termed the INPROGAS (Inflammatory PROtein-driven GAstric cancer Signature). When INPROGAS was tested in 22 independent samples, gastric mucosae were classified as “tumor” or “normal” with a sensitivity of 82% and a specificity of 72%. Although further tests are needed before this approach can be used in patients, the identification of a disease-specific biomarker panel early during gastric mucosa cancer development could facilitate more effective interventions against GC. Because the candidate biomarkers identified through antibody microarray-based oncoproteomics of the tumor-host GC microenvironment were found to mostly belong to the secreted class of proteins present in tissue and body fluids (*i.e.*, the secretome), our current findings may provide a great basis for non-invasive, blood-based identification of GC biomarkers for screening purposes. Thus, imbalances in the network of communication between cells in disease states may not only serve as a diagnostic indicator but could potentially reveal mechanistic insight into cancer development in the human stomach.

## METHODS

### Patients and tissue samples

This was a prospective, controlled, single blind analysis study. Pairs of GC and adjacent noncancerous mucosa were obtained after informed consent was received from patients (n=32) who underwent D2 gastrectomy (i.e., radical gastrectomy with level 2 extended lymphadenectomy) between January 2009 and July 2011 at the Hospital Universitari de Girona Dr. Josep Trueta in Girona, Catalonia, Spain. The study was reviewed and approved by the institutional review board and ethics committee. The prospective subject cohort consisted of matched pairs of tumor/normal gastric tissues from GC patients who fulfilled the following criteria: a) histological diagnosis of GC; b) any tumor node metastasis (TNM) stage; c) gastric resection with curative/radical intention; d) no chemotherapy or radiotherapy treatment prior to surgery; and e) signed informed consent. The clinicopathological data of the patients are summarized in Table 1.

**Table 1:** Patient Demographics and Gastric Cancer Characteristics

Clinicopathological characteristics	Patient number (%)
Age	
≤60 years	11 (34)
>60 years	21 (66)
Sex	
Male	18 (56)
Female	14 (44)
Histology	
Moderately differentiated adenocarcinoma	14 (44)
Poorly differentiated adenocarcinoma	18 (56)
Vascular invasion	
Yes	14 (44)
No	18 (56)
AJCC TNM stage*	
I	9 (28)
II	6 (19)
III	12 (37)
IV	5 (16)
Primary tumor	
T1	6 (19)
T2	12 (37)
T3	11 (34)
T4	3 (9)
Node status	
N0	12 (37)
N1	7 (22)
N2	8 (25)
N3	5 (16)
Metastasis	
M0	30 (94)
M1^a^	2 (6)

* According to the American Joint Committee on Cancer (AJCC)

a Peritoneal metastasis

**Table 2:** GC diagnostic test using INPROGAS The results are shown in modified 2 x 2 contingency tables that were used to calculate the percentage of classifications that agreed with the clinicopathological diagnosis. The values in parentheses represent 95% confidence intervals.

	Diseased	No-Disease	Totals		
Test POSITIVE	18 ^a^	6 ^b^	24	PPV ^g^ 75% [53-90]	LR-PT ^i^ 3 [1.5-6.1]
Test NEGATIVE	4 ^c^	16 ^d^	20	NPV ^h^ 80% [56-94]	LR-NT ^j^ 0.25 [0.0990-0624]
Totals	22	22			
	Sensitivity ^e^ 82% [53-90]	Specificity ^f^ 73% [49-89]			

a True Positive

b False Positive

c False Negative

d True Negative

e a/(a+c)

f d/(b+d)

g Positive Predictive Value = a/(a+b)

h Negative Predictive Value = d/(c+d)

i Likelihood Ratio Positive Test

j Likelihood Ratio Negative Test

[95% Confidence Interval] calculated with Binomial Expansion

To ensure the purity of the GC tissues, the specimens were excised from the cancerous cores. Thus, tumor samples of 3 x 3 x 5 mm3 were taken from areas without gross necrosis. Adjacent nontumor mucosa samples of 3 x 3 x 5 mm3 were taken from the same patient at a location 5 cm away from the tumor margin and were defined as the controls for each GC patient. The representative tumors and adjacent normal tissues of these patients were washed with physiological saline and subsequently frozen within 30 minutes of removal in a liquid nitrogen tank after immediate pathological examination. The senior pathologists routinely conducted the diagnosis for GC based upon Hematoxylin and Eosin (HE) staining. The TNM stage of the tumor was assigned according to the American Joint Committee on Cancer.

### Summary of experimental design

Thirty-two paired tumor and adjacent normal tissue samples were extracted, all of which were used to assess the expression level of more than 200 proteins with 5 different proteomic microarrays (see below). These 32 paired samples were divided into a training set (n=10), which was used to select the protein panel to distinguish between normal and GC tumor tissues of GC, and a validation set (n=22), which was used to confirm the ability of candidate proteins selected in the training set to classify blinded gastric samples as “GC” or “non-GC”.

### Proteomic chip-based analysis of protein expression in gastric mucosa

We employed protein arrays based on recently developed antibodies by RayBiotech (Norcross, GA, USA), which are capable of rapidly and specifically detecting the expression levels of numerous cytokines, growth factors, soluble receptors of growth factors, angiogenic factors, metalloproteinases and other proteins using small amounts of experimental samples in a single experiment. This technology is designed around the “sandwich immunoassay” principle. A panel of antibodies (capture antibodies) is immobilized at specific locations scored on the surface of a solid membrane, and incubation of the membrane arrays with biological samples results in the capture of soluble proteins by their corresponding antibodies. The bound proteins are detected by incubation with a cocktail of biotinylated antibodies, and corresponding signals are then visualized using enhanced chemiluminescent (ECL) techniques, colorimetry or infrared fluorescence. The following microarrays were used for each of the samples from patients enrolled in the study (i.e., tumor tissue lysates versus adjacent normal tissue lysates): RayBio® Human Cytokine Antibody Array C Series 1000 (Array VI + VII, which detects the expression of 120 cytokines in 2 membranes); RayBio® Human Angiogenesis Antibody Array C Series 1000 (Array 1 + Array 2, which detects the expression of 43 angiogenic factors in 2 membranes); RayBio® Human Matrix Metalloproteinases (MMPs) Antibody Array 1 (which detects the expression of 10 MMPs in a single membrane); RayBio® Human Growth Factor Antibody Array 1 (which detects the expression of 41 growth factors in a single membrane); and RayBio® Human Inflammation Antibody Array 3 (which detects the expression of 40 inflammatory factors in a single membrane). The information provided by the 5 proteomic microarrays was monitored in a quantitative manner (see below), and the expression levels of over 200 proteins were analyzed in each sample.

The protein test using antibody-based microarrays was performed according to the manufacturer's instructions. Briefly, prior to the start of the analysis, the membranes were blocked with 5% bovine serum albumin (BSA) in Tris-buffered saline (TBS; 0.01 M Tris HCl, pH 7.6, 0.15 M NaCl) for 1 h. After blocking to reduce the amount of non-specific binding, membranes were incubated with 750 μg total protein/tissue sample or 1 ml of serum for 2 h. After extensively washing the membranes with 0.1% Tween-20 in TBS (v/v) (i.e., 3 times for 5 minutes each) and TBS (2 times for 5 minutes each) to remove non-bound material, the membranes were incubated with a cocktail of biotin-labeled antibodies directed against the immobilized proteins by capture antibodies. Following antibody incubation, the membranes were washed as described above and subsequently incubated with horseradish peroxidase (HRP)-conjugated streptavidin (2.5 pg/ml) for 1 h at room temperature. Excess HRP-streptavidin was removed by washing in 0.1% TBS/Tween 20 and TBS. Finally, specific expression signals were detected using the ECL system.

The results obtained in each of the microarrays were evaluated using Analysis Tool Software, a data analysis program specifically designed for RayBio® Antibody Arrays. This analytical tool allows the user to perform the following tasks: a) locate the signal intensities (expression levels) in the antibody array map; b) provide a list of differentially expressed proteins (i.e., candidate markers); c) calculate mean signal intensities; d) analyze and subtract background data (i.e., noise); e) standardize data from different samples; and f) obtain comparative patterns of expression levels between different samples. This program operates based on Microsoft (MS) Excel computer software.

Ingenuity analysis. Signaling networks were constructed using Ingenuity Pathway Analysis (Ingenuity® Systems, Redwood City, CA, USA). Data sets containing identifiers of proteins that were significantly up- or downregulated were uploaded into the application. The ‘focus genes’ were then overlaid on the global molecular network developed from information in the Ingenuity Pathway Knowledge Base. Networks of these ‘focus genes’ (nodes) were algorithmically generated based on the principle that highly connected gene networks are the most biologically meaningful networks. All edges were supported by at least one reference from the literature stored in the Ingenuity Pathway Knowledge Base (the IPA interaction database is manually curated by scientists and updated quarterly). Briefly, the user-input or ‘focus genes’ list was compared to the ‘global molecular network’ (GMN) database consisting of thousands of genes and interactions. The focus genes were sorted based on highest to lowest connectivity within the GMN, and networks of approximately 35 genes were generated starting with the most connected focus gene. IPA assigns a p-value for a network of size n and an input focus gene list of size f by calculating the probability of finding f or more focus genes in a randomly selected set of n genes from the GMN. The intensity of the node color indicated the degree of expression (green scale for downregulated nodes; red scale for upregulated nodes). The nodes were displayed using various shapes, each of which represented a functional class of the gene products. The score indicated the likelihood of the genes in a network being found together due to random chance. Using a 99% confidence interval, scores of ≥ 3 were deemed significant.
